# PBDEs and Cryptorchidism: Main et al. Respond

**DOI:** 10.1289/ehp.11052R

**Published:** 2008-05

**Authors:** Katharina M. Main, Jorma Toppari

**Affiliations:** University Department of Growth and Reproduction, Copenhagen, Denmark, E-mail: katharina.main@rh.regionh.dk; Departments of Physiology and Paediatrics, University of Turku, Turku, Finland

In their letter, Banasik et al. emphasize that the etiology of cryptorchidism may be multifactorial, and we agree. As stated in the “Discussion” of our article (Main et al. 2007), exposure to environmental chemicals is only one of many adverse factors that alone, or in combination with each other, may cause testicular maldescent. In turn, lifestyle factors such as smoking and drinking of alcohol may be interrelated, and a certain lifestyle may augment the risk of exposure to endocrine-disrupting chemicals. Genetic factors, complex medical syndromes, and lifestyle factors have been previously identified as risk factors for cryptorchidism, but a significant proportion of cryptorchidism cases still remains unexplained (Virtanen et al. 2007).

In our study (Main et al. 2007) we found an association between the level of polybrominated diphenyl ethers (PBDEs) in breast milk and congenital cryptorchidism. We reported that this remained significant after adjusting for other well-known risk factors for cryptorchidism, such as premature birth or low birth weight for gestational age (Main et al. 2007), as well as adjusting for maternal age, maternal prepregnancy body mass index, parity, and date of childbirth within the cohort, which may affect the level of compounds found in breast milk samples (Main et al. 2007). In the analysis of the entire Danish–Finnish binational cohort, other novel risk factors for cryptorchidism, such as regular maternal alcohol consumption during pregnancy and mild diabetes, were identified (Damgaard et al. 2007; (Virtanen et al. 2006).

We originally decided against including additional confounders in our analysis (Main et al. 2007), considering the size of our study group and thus the low number of cases for each confounder, which induces a potential risk of both false-positive and false-negative results (Table 1). However, in a binary logistic regression analysis including all confounders, as suggested by Banasik et al., the association between the level of the seven most prevalent PBDEs (BDEs 28, 47, 66, 99, 100, 153, and 154) and congenital cryptorchidism remained significant (*p* < 0.039). Confounders included maternal age, maternal prepregnancy body mass index, gestational age, and weight for gestational age included as continuous variables; parity (1 vs. 2 vs. ≥ 3), country of origin (Denmark/Finland), social class (high- and low-grade professionals vs. skilled and unskilled workers vs. students and unemployed), smoking (yes/no), diabetes (yes/no), binge episodes (yes/no), and alcohol consumption (0 vs. 1–4 vs. ≥ 5 drinks/week) were entered as categorical factors, as described by Damgaard et al. (2007).

In conclusion, the association between perinatal exposure to PBDEs and congenital cryptorchidism was significant even after controlling for confounding factors. Our study could not determine whether a direct causal relationship exists or whether other factors contribute to our findings. Because the exposure to PBDEs is still considerable in some areas, our results should raise concern and stimulate further investigations of human populations.

## Figures and Tables

**Table 1:** Additional characteristics (number) of the study population who provided breast milk samples.

	Denmark		Finland	
Characteristic	Control (*n* = 36)	Cryptorchid (*n* = 29)	Control (*n* = 32)	Cryptorchid (*n* = 33)
Delivery type				
Vaginal delivery	28	16	26	23
Vacuum extraction	6	6	2	4
Cesarean section	2	7	4	4
Alcohol comsumption (drinks/week)				
0	13	16	26	27
1–4	20	9	6	6
≥ 5	3	4	0	0
Alcohol binge-drinking episodes				
Yes	2	2	2	0
No	33	25	28	29
Social class				
High- and low-grade professionals	20	16	17	21
Skilled and unskilled workers	9	7	9	6
Students and unemployed	7	6	6	5

Missing data were treated as missing values in the statistical analysis.
